# Supplementary material for: Recruitment of a splicing factor to the nuclear lamina for its inactivation
Source: Commun Biol. 2022 Jul 22;5:736. doi: 10.1038/s42003-022-03689-y (PMC9307855; doi:10.1038/s42003-022-03689-y)
Supplement: Supplementary file 2 — Supplementary Information [file 42003_2022_3689_MOESM2_ESM.pdf]

# Supplementary Data

# Recruitment of a splicing factor to the nuclear lamina for its inactivation

Karen Vester[1,*], Marco Preußner[2], Nicole Holton[1], Suihan Feng[3], Carsten Schultz[3], Florian Heyd[2], Markus C. Wahl[1,4,*]

[1] Freie Universität Berlin, Institute of Chemistry and Biochemistry, Laboratory of Structural Biochemistry, Takustrasse 6, D-14195 Berlin, Germany

[2] Freie Universität Berlin, Institute of Chemistry and Biochemistry, Laboratory of RNA Biochemistry, Takustrasse 6, D-14195 Berlin, Germany

[3] Oregon Health and Science University, Department of Chemical Physiology and Biochemistry, 3181 SW Sam Jackson Park Rd., L334, Portland, OR 97239, USA

[4] Helmholtz-Zentrum Berlin für Materialien und Energie, Macromolecular Crystallography, Albert-Einstein-Straße 15, D-12489 Berlin, Germany

* Correspondence to: karenvester@zedat.fu-berlin.de; markus.wahl@fu-berlin.de

# Supplementary Tables

## Supplementary Table S1. Primers[(a)]

| Probed event | Forward | Reverse |
|---|---|---|
| Insertion of FKBP-coding region on *prpf38A* locus; red[(b)] | CAGAGAACGTCCTCAGTTTC | GTACTTGGACTCATAGATTCGC |
| Insertion of FKBP-coding region on *prpf38A* locus; blue[(b)] | CAAACTGACTATATCTCCAGATTATG | CTTATATTAGCTAGTGAACATTGCAC |
| Insertion of FKBP-coding region on *prpf38A* locus; cyan[(b)] | GTTGAAGGTCTTTAAGGACCTAAC | CTAGAAATGAGATACATTGTGTACAC |
| *prpf38A* first exon (E1) | GAACCTGGATGTGAGGCATTAAAG | GTCCAAAGCACTCCTCTTTCC |
| Stable integration of lamin A-ECFP-SNAP-coding region; blue[(c)] | CAGGAGCTCAATGATCGCTTG | CCAGCTCCTTCTTATACTGCTC |
| Stable integration of lamin A-ECFP-SNAP-coding region; cyan[(c)] | GCACAAGCTGGAGTACAACTAC | CCTCGAGTTTAAACGCGGATC |
| *fam90A1* pre-mRNA splicing | GAAGCTCTGAAGATGGATCATG | GATGCAGCATCCAGGAAGAC |
| *cdc6* pre-mRNA splicing | CTCTTCAGCAGAAGATCTTG | GAAAGTGACAAACACTCTGAC |
| *letm2* pre-mRNA splicing | GTAAAGTTGGAACTAGCAAAATTTC | GAACTATCTCCTTTGTGCTG |

[a], all sequences 5'-to-3'.
[b], colors refer to primer symbols in Figure 4A and Figure 5A, upper scheme.
[c], colors refer to primer symbols in Figure 5A, lower scheme.

**Supplementary Table S2. Guide RNA coding sequences[(a)]**

| gRNA | Sequence |
|---|---|
| gRNA1 | AATGGCTAACCGTACAGTGA |
| gRNA2 | TGTGCGCATCCTTCACTGTA |
| gRNA3 | GCCTTCAATTCTATTTCCGT |
| gRNA4 | TCCGACGGAAATAGAATTGA |
| ctr_gRNA1 | AAAGAAAACTTCGCGCCAAT |
| ctr_gRNA2 | AGAAATGAAAAGAAGGTGGA |

[a], all sequences 5’-to-3’.

## Supplementary Figures

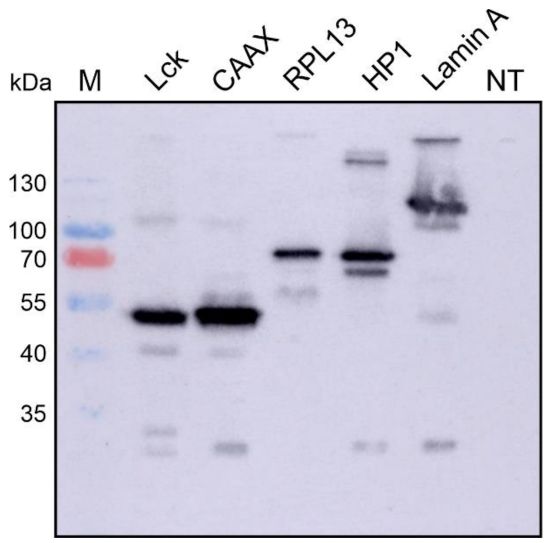


**Supplementary Figure S1. Western-Blot of transfected anchors.**

HEK293T cells were transfected with ECFP-SNAP-tagged anchor constructs (Lck, Lck-ECFP-SNAP; CAAX, ECFP-SNAP-CAAX; RPL13, RPL13-ECFP-SNAP; HP1, HP1-ECFP-SNAP; Lamin A, lamin A-ECFP-SNAP; NT, no treatment control). The lysates, harvested two days after transfection, were tested for expression with an anti-GFP antibody, which also recognizes the closely related ECFP. The blot shows the expected sizes for the anchor protein constructs.

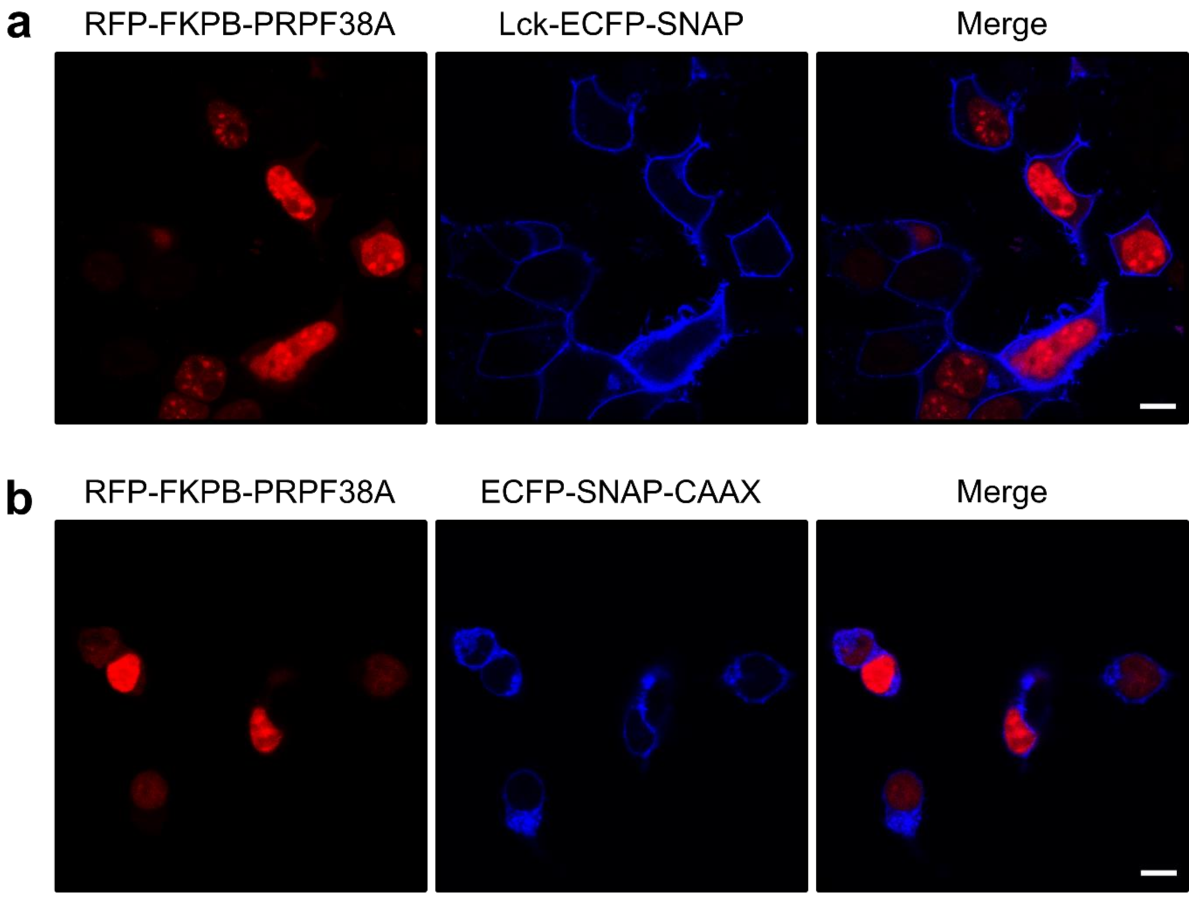


**Supplementary Figure S2. Long-time recruitment trial to the PM.**

HEK293T cells were transfected with vectors expressing RFP-FKBP-PRPF38A (red channel; left) and Lck-ECFP-SNAP (blue channel; middle) and treated with rCD1 for 24 hours (**a**), or were transfected with vectors expressing RFP-FKBP-PRPF38A (red channel; left) and ECFP-SNAP-CAAX (blue channel; middle) and treated with rCD1 for 24 hours (**b**). No co-localization was observed (merged images; right). Scale bars (10 μm) are shown as white lines on the bottom right of the merged images.

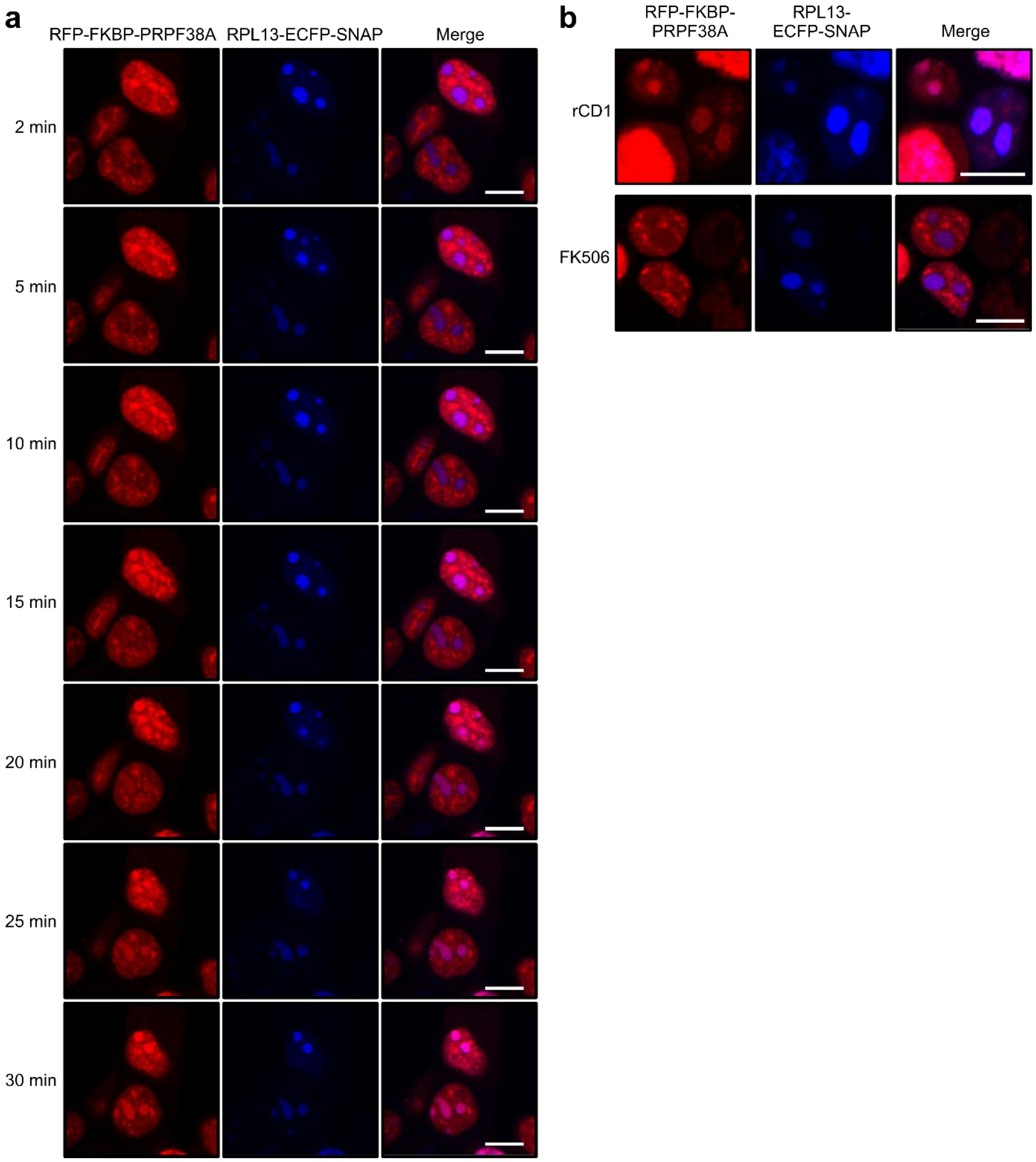


**Supplementary Figure S3. Time-dependent recruitment to RPL13-ECFP-SNAP.**

The recruitment of RFP-FKBP-PRPF38A to RPL13-ECFP-SNAP was observed in HEK293T cells co-transfected with both constructs. After rCD1 addition, dimerizarion was monitored over time, showing a time-dependent increase in co-localization (**a**). After rCD1-based dimerization for more than 30 minutes (**b**, upper image), FK506 was added and a complete reversal of the

dimerization was observed 5 minutes after FK506 addition (**b**, lower image). Scale bars (10 μm) are shown as white lines on the bottom right of the merged images.

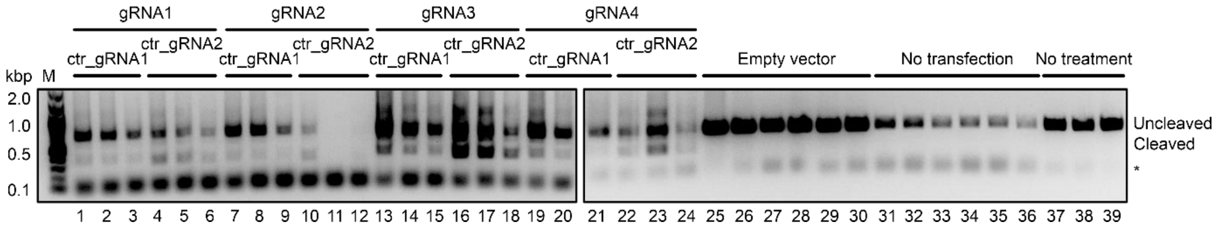


**Supplementary Figure S4. Cleavage efficiency test of the gRNAs.**

Ethidium bromide-stained agarose gels showing results of PCR analyses of genomic DNA extracted from cells co-transfected with the four indicated gRNAs and the indicated control (ctr) gRNAs to test for the cleavage efficiency. Cleavage is revealed by a deletion of approximately 300 bp as indicated on the right. The gels show cleavage tests at three serial cell dilutions (1:1, lanes 1, 4, 7, 10, 13, 16, 19, 22, 25, 28, 31, 34, 37; 1:1.67, lanes 2, 5, 8, 11, 14, 17, 20, 23, 26, 29, 32, 35, 38; 1:2.67, lanes 3, 6, 9, 12, 15, 18, 21, 24, 27, 30, 33, 36, 39). Cells transfected with empty PX459 2.0 vector and treated with puromycin (lanes 25-30), non-transfected cells treated with puromycin (lanes 31-36; not viable after treatment) and untreated cells (lanes 37-39) were loaded as controls and do not show the lower band corresponding to the cleaved DNA. M, size marker (kbp); *, primers and/or primer dimers.

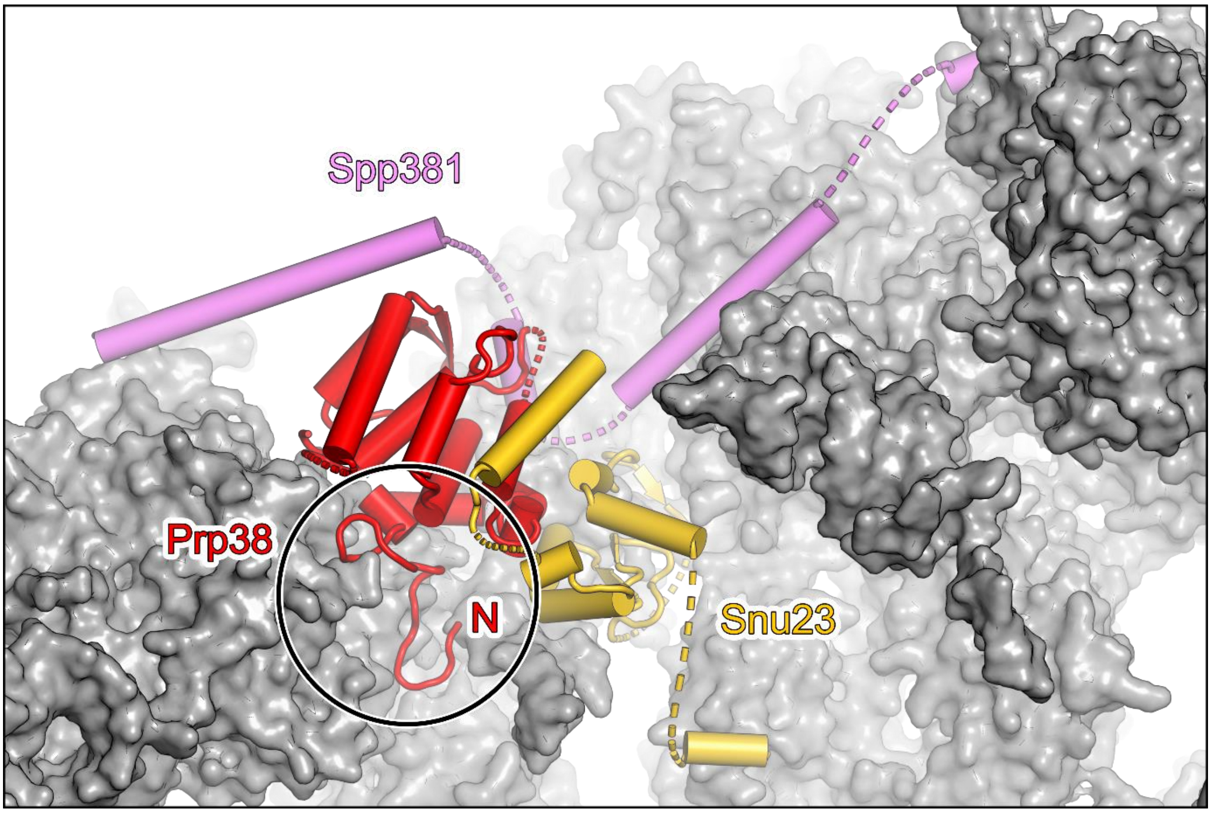


**Supplementary Figure S5. Prp38 in a cryoEM structure of the yeast spliceosomal B complex.**

Cartoon plot of Prp38 (yeast PRPF38A ortholog; red) with the closest interaction partners, Spp381 (yeast MFAP ortholog [1]; violet) and Snu23 (gold) from a yeast spliceosomal B complex structure (PDB ID 5NRL) [2]. Other portions of the spliceosome are shown as a gray surface. Approximately 20 amino acid residues of the N-terminal part of Prp38 (N; circled) are irregularly structured.

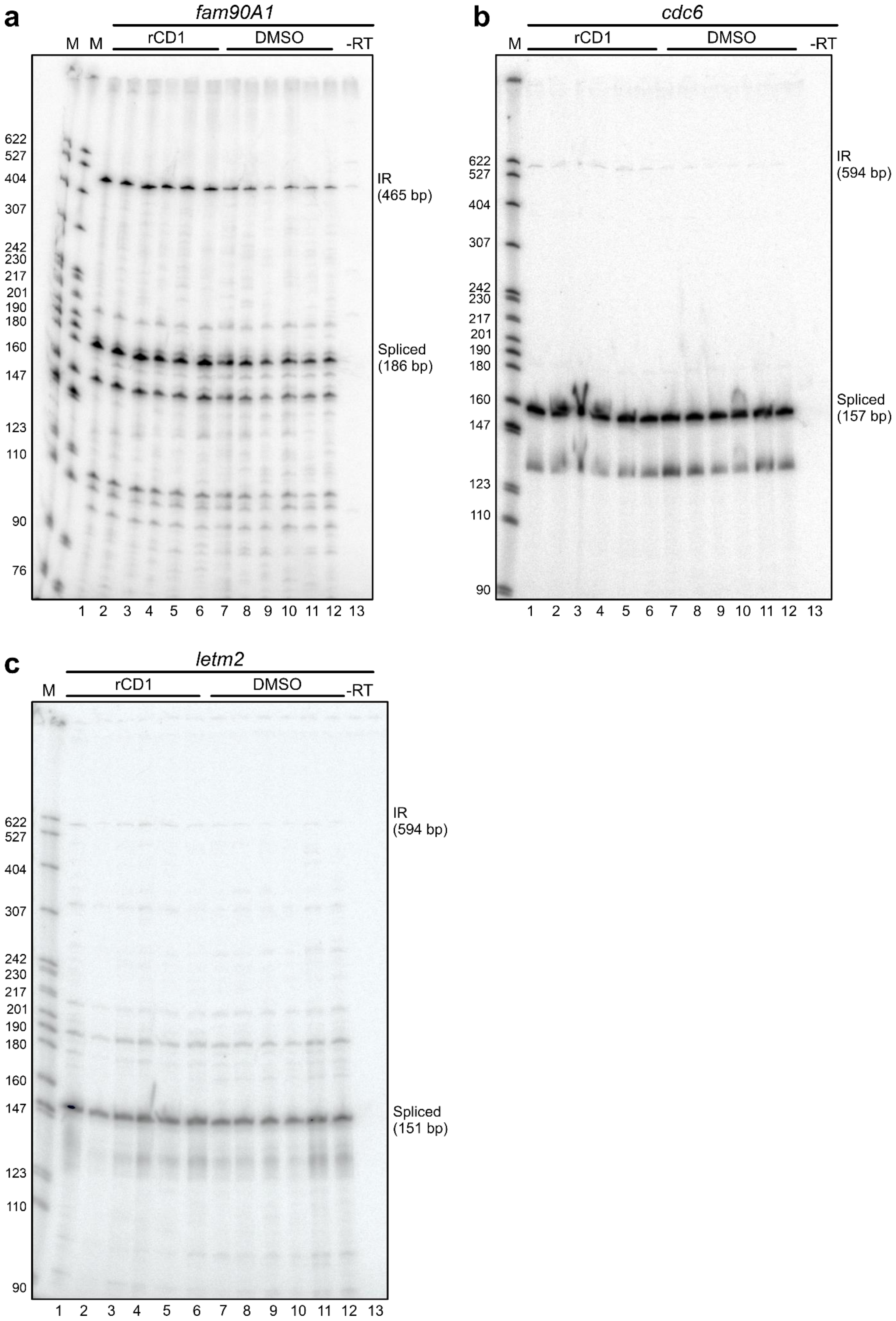

a
fam90A1
M M
rCD1
DMSO
-RT
622
527
404
307
242
230
217
201
190
180
160
147
123
110
90
76
IR
(465 bp)
Spliced
(186 bp)
1 2 3 4 5 6 7 8 9 10 11 12 13
b
cdc6
M
rCD1
DMSO
-RT
622
527
404
307
242
230
217
201
190
180
160
147
123
110
90
IR
(594 bp)
Spliced
(157 bp)
1 2 3 4 5 6 7 8 9 10 11 12 13
c
letm2
M
rCD1
DMSO
-RT
622
527
404
307
242
230
217
201
190
180
160
147
123
110
90
IR
(594 bp)
Spliced
(151 bp)
1 2 3 4 5 6 7 8 9 10 11 12 13

**Supplementary Figure S6. Radioactive PCR analyses.**

(**a-c**) Complete gel images of panels in Figure 6d. PCR analyses of six biological replicates were performed with radioactively labeled forward primers annealing to the upstream exon and unlabeled primers annealing to the respective downstream exons of the *fam90A1* (**a**), *cdc6* (**b**) or *letm2* (**c**) genes in doubly engineered FKBP-PRPF38A CRISPR/lamin A-ECFP-SNAP stable cells, showing enhanced intron retention after rCD1 treatment (lanes 1-6) compared to DMSO-treated cells (lanes 7-12). -RT, PCR analysis without addition of reverse transcriptase upon cDNA generation (control for the background generated from undigested genomic DNA). The nature of the PCR products and their sizes are indicated on the right. IR, intron-retained product.

## Supplementary References


1 Ulrich, A. K. & Wahl, M. C. Human MFAP1 is a cryptic ortholog of the Saccharomyces cerevisiae Spp381 splicing factor. *BMC Evol Biol* **17**, 91, doi:10.1186/s12862-017-0923-1 (2017).

2 Plaschka, C., Lin, P. C. & Nagai, K. Structure of a pre-catalytic spliceosome. *Nature* **546**, 617-621, doi:10.1038/nature22799 (2017).
