# Supplementary material for: Recruitment of a splicing factor to the nuclear lamina for its inactivation
Source: Commun Biol. 2022 Jul 22;5:736. doi: 10.1038/s42003-022-03689-y (PMC9307855; doi:10.1038/s42003-022-03689-y)
Supplement: Supplementary file 3 — Description of Additional Supplementary Files [file 42003_2022_3689_MOESM3_ESM.pdf]

Description of Additional Supplementary Files

File name: Supplementary Data 1
Description: Excel file including Western blots and gel images as well as the raw data used for quantifications, which are displayed in graphs and box-plots in the main text.
