# Supplementary material for: Recruitment of a splicing factor to the nuclear lamina for its inactivation
Source: Commun Biol. 2022 Jul 22;5:736. doi: 10.1038/s42003-022-03689-y (PMC9307855; doi:10.1038/s42003-022-03689-y)
Supplement: Supplementary file 5 — Reporting Summary [file 42003_2022_3689_MOESM5_ESM.pdf]

nature portfolio

Corresponding author(s): Markus C. Wahl

Last updated by author(s): Jun 21, 2022

Reporting Summary

Nature Portfolio wishes to improve the reproducibility of the work that we publish. This form provides structure for consistency and transparency in reporting. For further information on Nature Portfolio policies, see our Editorial Policies and the Editorial Policy Checklist.

Statistics

For all statistical analyses, confirm that the following items are present in the figure legend, table legend, main text, or Methods section.

n/a	Confirmed	
☐	☒	The exact sample size (*n*) for each experimental group/condition, given as a discrete number and unit of measurement
☐	☒	A statement on whether measurements were taken from distinct samples or whether the same sample was measured repeatedly
☐	☒	The statistical test(s) used AND whether they are one- or two-sided<br>*Only common tests should be described solely by name; describe more complex techniques in the Methods section.*
☒	☐	A description of all covariates tested
☒	☐	A description of any assumptions or corrections, such as tests of normality and adjustment for multiple comparisons
☒	☐	A full description of the statistical parameters including central tendency (e.g. means) or other basic estimates (e.g. regression coefficient) AND variation (e.g. standard deviation) or associated estimates of uncertainty (e.g. confidence intervals)
☒	☐	For null hypothesis testing, the test statistic (e.g. *F*, *t*, *r*) with confidence intervals, effect sizes, degrees of freedom and *P* value noted<br>*Give P values as exact values whenever suitable.*
☒	☐	For Bayesian analysis, information on the choice of priors and Markov chain Monte Carlo settings
☒	☐	For hierarchical and complex designs, identification of the appropriate level for tests and full reporting of outcomes
☒	☐	Estimates of effect sizes (e.g. Cohen's *d*, Pearson's *r*), indicating how they were calculated

Our web collection on statistics for biologists contains articles on many of the points above.

Software and code

Policy information about availability of computer code

Data collection	Leica Application Suite X (LAS X)
Data analysis	Prism Version 5, analysis and graphing software, GraphPad<br>ImageQuant, version 5.2, image analysis software, Cytiva<br>Fiji (Schindelin, J., Arganda-Carreras, I., Frise, E., Kaynig, V., Longair, M., Pietzsch, T., … Cardona, A. (2012). Fiji: an open-source platform for biological-image analysis. Nature Methods, 9(7), 676–682. doi:10.1038/nmeth.2019)

For manuscripts utilizing custom algorithms or software that are central to the research but not yet described in published literature, software must be made available to editors and reviewers. We strongly encourage code deposition in a community repository (e.g. GitHub). See the Nature Portfolio guidelines for submitting code & software for further information.

Data

Policy information about availability of data

All manuscripts must include a data availability statement. This statement should provide the following information, where applicable:

- Accession codes, unique identifiers, or web links for publicly available datasets
- A description of any restrictions on data availability
- For clinical datasets or third party data, please ensure that the statement adheres to our policy

RNAseq data were deposited Gene Expression Omnibus (https://www.ncbi.nlm.nih.gov/geo) under accession code GSE182412. All other data supporting the findings of this study are described in the manuscript or in the Supplementary Data or are available from the corresponding authors on request.

Field-specific reporting

Please select the one below that is the best fit for your research. If you are not sure, read the appropriate sections before making your selection.

☒ Life sciences ☐ Behavioural & social sciences ☐ Ecological, evolutionary & environmental sciences

For a reference copy of the document with all sections, see nature.com/documents/nr-reporting-summary-flat.pdf

Life sciences study design

All studies must disclose on these points even when the disclosure is negative.

Sample size	Sample size of at least three replicates was used to indicate an appropriate standard deviation of the measurements.
Data exclusions	For quantification of the recruitment timescale of cells overexpressing target and anchor constructs (figure 2b), only cells expressing both constructs according to confocal images were used for quantification as ROIs. Cells only expressing one construct were excluded from quantification.
Replication	-for recruitment images, different technical and biological replicates were prepared<br>-RNAseq data was based on three replicates<br>-validation of RNAseq data was done in biological replicates
Randomization	n.a.
Blinding	The sample IDs/ treatments were kept from preparation of the samples throughout the analysis.

Reporting for specific materials, systems and methods

We require information from authors about some types of materials, experimental systems and methods used in many studies. Here, indicate whether each material, system or method listed is relevant to your study. If you are not sure if a list item applies to your research, read the appropriate section before selecting a response.

Materials & experimental systems

n/a	Involved in the study	
☐	☒	Antibodies
☐	☒	Eukaryotic cell lines
☒	☐	Palaeontology and archaeology
☒	☐	Animals and other organisms
☒	☐	Human research participants
☒	☐	Clinical data
☒	☐	Dual use research of concern

Methods

n/a	Involved in the study	
☒	☐	ChIP-seq
☒	☐	Flow cytometry
☒	☐	MRI-based neuroimaging

Antibodies

Antibodies used	Antibodies against Prpf38a (Novus Biologicals; NBP2-37697, NBP2-33673); antibody against GFP and related variants (Santa Cruz; sc-9996); antibody against Actin (Merck Millipore; MAB1501); goat anti-rabbit antibody (Thermo Fisher Scientific; 31460) and goat anti-mouse antibody (Thermo Fisher Scientific; 31430).
Validation	Novus Biologicals; NBP2-37697<br>https://www.novusbio.com/products/prpf38a-antibody_nbp2-37697<br>Novus Biologicals; NBP2-33673<br>https://www.fishersci.com/shop/products/anti-prpf38a-polyclonal-novus-biologicals-1/NBP233673<br>Santa Cruz; sc-9996<br>https://www.scbt.com/de/p/gfp-antibody-b-2<br>Merck Millipore; MAB1501<br>https://www.merckmillipore.com/DE/de/product/Anti-Actin-Antibody-clone-C4,MM_NF-MAB1501

Eukaryotic cell lines

Policy information about cell lines

Cell line source(s)	HEK293T

Authentication	The parental cell line was derived from the Leibniz-Institut DSMZ-Deutsche Sammlung von Mikroorganismen und Zellkulturen GmbH, Braunschweig, Deutschland
Mycoplasma contamination	The cell lines were tested for mycoplasma contamination and confirmed to be negative
Commonly misidentified lines (See ICLAC register)	n/a
